# Global transcriptional profiling of *Burkholderia pseudomallei *under salt stress reveals differential effects on the Bsa type III secretion system

**DOI:** 10.1186/1471-2180-10-171

**Published:** 2010-06-14

**Authors:** Pornpan Pumirat, Jon Cuccui, Richard A Stabler, Joanne M Stevens, Veerachat Muangsombut, Ekapot Singsuksawat, Mark P Stevens, Brendan W Wren, Sunee Korbsrisate

**Affiliations:** 1 Department of Immunology, Faculty of Medicine Siriraj Hospital, Mahidol University, Bangkok 10700, Thailand; 2 Department of Infectious and Tropical Diseases, London School of Hygiene and Tropical Medicine, London WC1E 7HT, UK; 3 Division of Microbiology, Institute for Animal Health, Compton, Berkshire RG20 7NN, UK

## Abstract

**Background:**

*Burkholderia pseudomallei *is the causative agent of melioidosis where the highest reported incidence world wide is in the Northeast of Thailand, where saline soil and water are prevalent. Moreover, recent reports indicate a potential pathogenic role for *B. pseudomallei *in cystic fibrosis lung disease, where an increased sodium chloride (NaCl) concentration in airway surface liquid has been proposed. These observations raise the possibility that high salinity may represent a favorable niche for *B. pseudomallei*. We therefore investigated the global transcriptional response of *B. pseudomallei *to increased salinity using microarray analysis.

**Results:**

Transcriptome analysis of *B. pseudomallei *under salt stress revealed several genes significantly up-regulated in the presence of 320 mM NaCl including genes associated with the *bsa*-derived Type III secretion system (T3SS). Microarray data were verified by reverse transcriptase-polymerase chain reactions (RT-PCR). Western blot analysis confirmed the increased expression and secretion of the invasion-associated type III secreted proteins BipD and BopE in *B. pseudomallei *cultures at 170 and 320 mM NaCl relative to salt-free medium. Furthermore, salt-treated *B. pseudomallei *exhibited greater invasion efficiency into the lung epithelial cell line A549 in a manner partly dependent on a functional Bsa system.

**Conclusions:**

*B. pseudomallei *responds to salt stress by modulating the transcription of a relatively small set of genes, among which is the *bsa *locus associated with invasion and virulence. Expression and secretion of Bsa-secreted proteins was elevated in the presence of exogenous salt and the invasion efficiency was enhanced. Our data indicate that salinity has the potential to influence the virulence of *B. pseudomallei*.

## Background

*Burkholderia pseudomallei *is a saprophyte and the causative agent of melioidosis, a human infectious disease endemic in some tropical areas including southeast Asia and northern Australia [1]. Inhalation is a recognized route of infection with this organism and pulmonary disease is common [1,2]. Owing to its aerosol infectivity, the severe course of infection, and the absence of vaccines and fully effective treatments, *B. pseudomallei *is classified as a hazard category three pathogen and considered a potential biothreat agent [2]. *B. pseudomallei*, is a Gram negative bacillus found in soil and water over a wide endemic area and mainly infects people who have direct contact with wet soil [1,3]. In Thailand, the highest incidence of melioidosis is in the northeast region, at a rate of approximately 3.6-5.5 per 100,000 human populations annually. Septicaemic presentation of disease is associated with a high mortality rate (up to 50% in adults and 35% in children) [4]. A remaining enigma is that *B. pseudomallei *is commonly present in this region of Thailand, but rarely found in other parts of the country or indeed other parts of the world [5,6]. Of potential significance is the abundance of enclosed bodies of water with a high salt content and saline soils in the northeast region of Thailand [7]. The electrical conductivity of salt-affected soil in Northeast Thailand is ranging between 4 to 100 dS/m, which is higher than normal soil from other parts of Thailand (approximately 2 dS/m) (Development Department of Thailand). An increase in salt concentration in these regions is believed to be caused both by natural phenomena and man-made activities [7]. One may speculate that the organism has developed an ability to thrive in saline conditions and as such has gained a selective ecological advantage over other soil dwelling micro organisms.

Previously, it has been indicated that the killing efficiency of *Burkholderia *species, including *B. pseudomallei *against the nematode *Caenorhabditis elegans *was enhanced in a high osmolarity conditions [8]. This putative link between high salt concentration and an ability to withstand such conditions is evident in a subset of closely related organisms, namely, the *B. cepacia *complex (BCC). These are opportunistic pathogens of cystic fibrosis (CF) sufferers [9,10] where the lung airway surface liquid has been hypothesized an increased concentration of NaCl [11], that is typically 2-fold higher than in healthy lungs [12]. More recently, reports of a potential pathogenic role for *B. pseudomallei *in CF lung disease have been made [13]. To date, little is known of how elevated NaCl concentrations affect *B. pseudomallei*.

As *B. pseudomallei *can survive and multiply under different environmental conditions and in various hosts [14,15], it is likely that this organism has developed strategies to cope with high salt concentrations in both the natural environment and in its respective hosts. In the river water environment, osmolarity is believed to be less than 60 mM NaCl whilst in the human lung it is normally 50 to 100 mM and in the blood the bacterium can encounter a concentration of up to 150 mM NaCl [11,16]. Recently, the secreted protein profile of *B. pseudomallei *following growth in salt-rich medium was revealed and provided a clue to the adaptive response of the organism to this stress [17]. Increased secretion of several metabolic enzymes, stress response protein GroEL, beta-lactamase like proteins and potential virulence factors were noted. Moreover, the effects of increasing salt concentration on the expression of a number of genes within the organism *B. cenocepacia*, formerly *B. cepacia *genomovar III, a close relative of *B. pseudomallei *have been described [18]. Genes found to be upregulated included an integrase, an NAD-dependent deacetylase and an oxidoreductase amongst others. In *Pseudomonas aeruginosa*, another close relative of *B. pseudomallei*, the up-regulation of genes associated with osmoprotectant synthesis, putative hydrophilins, and a Type III protein secretion system (T3SS) after growth under steady-state hyperosmotic stress has been demonstrated [19]. High salt stress was also demonstrated to be one of the environmental stimuli affecting expression of the Ysa T3SS in *Yersinia enterocolitica *[20,21]. The *B. pseudomallei *strain K96243 genome encodes three predicted T3SSs, one related to the Inv/Mxi-Spa systems of *Salmonella *and *Shigella *(Bsa, T3SS-3) and two related to systems found in plant bacterial pathogens (T3SS-1 and -2). Of these, only the Bsa system appears to play a significant role in virulence in rodent models of melioidosis [22,23], likely as a consequence of its effects on invasion, endosome escape and intracellular survival [24,25]. It is noteworthy that transcription of the invasion-associated *Salmonella *pathogenicity island-1 genes homologous to the *bsa *locus is activated by the addition of NaCl [26]. Gaining an understanding of the ability of *B. pseudomallei *to survive in the presence of high salt concentrations is therefore significant, as this may provide insights into its pathogenicity and persistence in endemic areas.

Here we used a genome-wide oligonucleotide microarray to quantify the transcription of *B. pseudomallei *genes in response to salt stress. Differential regulation of a subset of genes was confirmed by RT-PCR and by analysis of production of the encoded proteins. Our data reveal that exogenous NaCl induces the virulence-associated Bsa T3SS and the consequences of such for invasion of A549 cells were investigated.

## Results

### *B. pseudomallei *growth was inhibited in high salt

**Figure 1:**
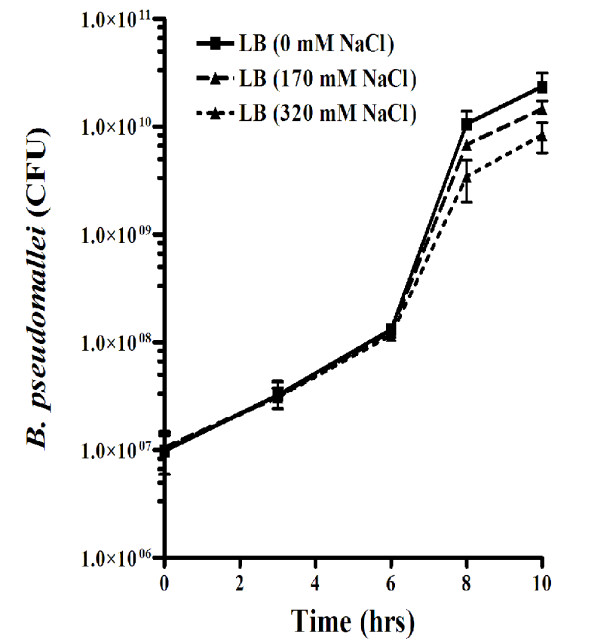
**Growth kinetics of *B. pseudomallei***. *B. pseudomallei *K96243 growth in LB broth containing 0, 170 or 320 mM NaCl was determined by colony plate counting. The data points and error bars represent mean colony forming unit (CFU) and standard deviation from triplicate experiments.

To better understand the physiology of *B. pseudomallei *in response to elevated salt, we titrated the effect of salt on *B. pseudomallei *growth starting from salt-free Luria Bertani (LB) medium and standard LB medium containing 170 mM plus various concentrations of NaCl (170+150, 170+300 and 170+450 mM), and found that conditions with 470 and 620 mM NaCl had severe impairment on *B. pseudomallei *growth (data not shown). For lower NaCl concentrations, the growth kinetics of *B. pseudomallei *K96243 cultured in standard LB medium containing 170 or 320 mM NaCl was similar until 6 hrs; the growth rate thereafter was impaired when cultured in LB broth containing 320 mM NaCl (Figure 1). The doubling time in NaCl-supplemented LB broth was calculated to be 53 ± 4.3 min compared to 38 ± 3.0 min in standard LB broth (*t*-test; *P value *= 0.027). In addition, we found that growth of *B. pseudomallei *in salt-free medium was faster than in standard LB medium supplemented with 170 and 320 mM NaCl (Figure 1). This data indicated that increased NaCl reduced the logarithmic growth rate of *B. pseudomallei*.

### Differential transcriptome of *B. pseudomallei *during growth in high salt

Our studies indicated that growth of *B. pseudomallei *was severely impaired during culture at NaCl concentrations of 470 and 620 mM (data not shown). This suggested that these concentrations may be too high to detect salt-specific transcriptional changes. A previous study carried out in our laboratory demonstrated a significantly altered secretome when the organism was grown in 320 mM NaCl compared to standard LB medium (170 mM NaCl) [16]. We therefore chose 170 and 320 mM NaCl conditions for further investigation by microarray analysis. We elected to isolate RNA from cultures at 3 and 6 hrs for transcriptome analysis because no significant difference in bacterial growth survival was noted at these time points (Figure 1). RNA was stabilized and extracted immediately and analyzed for differential gene expression by hybridisation to a *B. mallei/pseudomallei *whole genome 70 mer oligonucleotide microarray version 2 (a kind gift from the J. Craig Venter Institute) which containing 9,826 reporters based on the *B. mallei *ATCC 23344, *B. mallei *GB8 Horse 4, *B. pseudomallei *1710b and *B. pseudomallei *K96243 genome. Four biological replicates generated for each sample clustered together indicating minimal experimental variation (Additional file 1). ANOVA statistical analysis and multiple testing correction identified 10 genes as significantly altered in their transcription (Table 1). Among the salt-regulated genes of *B. pseudomallei *identified in this study were a putative two-component system response regulator, bacterial metabolic enzymes, and hypothetical proteins. Fold changes of altered genes at both 3 and 6 hrs ranged from 1.1-1.8 and 1.1-26.6, respectively. Noticeably, a larger dynamic range of gene expression was observed after 6 hrs cultures, with the majority of the 10 genes being up-regulated.

**Table 1:** Effect of NaCl treatment on transcription of *B. pseudomallei *K96243 genes as detected by microarray analysis.

Putative function	Gene	Fold change		*P value*
			
		3 hrs	6 hrs	
Formyltetrahydrofolate deformylase	BPSL0543	1.3*	-1.1	0.037
Putative adenylate cyclase	BPSL3054	1.5*	-1.0	0.038

Acyl-CoA dehydrogenase domain protein	BPSS1272	1.0	4.4*	0.035
Hypothetical protein	BPSS2215	-1.2	7.3*	0.038
Hypothetical protein	BPSS2221	1.0	3.0*	0.037
Response regulator	BPSS2231	-1.4	6.4*	0.038
Hypothetical protein	BPSS2232	1.1	26.6*	0.037
Hypothetical protein	BPSS2240	-1.8	6.8*	0.038
Short chain dehydrogenase/oxidoreductase	BPSS2242	1.0	10.0*	0.035
Glycosyltransferase family 9 protein	BPSS2255	1.0	2.6*	0.037

* Genes showed mean significant differences comparing between standard LB medium (170 mM) and LB with 320 mM NaCl using ANOVA with a Benjamini-Hochberg multiple testing correction (*P *value < 0.05).

Due to the stringent statistic analysis by ANOVA and false discovery rate correction, it is possible that potentially significant trends were masked. Owing to the effect of salt on loci encoding T3SS in *Pseudomonas*, *Yersinia *and *Salmonella*, we examined the microarray data for effects on predicted Type III secretion-associated loci by only looking at the test ratio and standard deviation (SD) and computing a confidence of that data point using a standard two tailed *t*-test (Table 2). Interestingly, a number of *bsa*-derived T3SS genes were found to have altered expression levels during culture in LB broth containing 320 mM NaCl compared to standard LB at 3 hrs and 6 hrs (*t*-test; *P value *< 0.05) (Table 2 and Additional file 2), in particular those encoding predicted secreted effectors and translocon components. We also found that the expression of beta-lactamase family protein (BPSS2119) and GroEL (BPSS0477) was upregulated in LB containing 320 mM NaCl by approximately 1.2 fold compared with those in standard LB broth at the 6 hrs time point (*t*-test; *P *value < 0.05) (Additional file 3). In contrast genes encoding for T3SS-1 and T3SS-2 (except BPSS1603 and BPSS1617) did not show a significant difference in expression levels (*t*-test; *P *value > 0.05) (Additional file 3).

**Table 2:** Effect of NaCl on transcription of genes associated with the *bsa*-derived T3SS in *B. pseudomallei *K96243.

Putative function	Gene	Fold change	
		
		3 hrs	6 hrs
**Type III structural proteins**			
BsaZ	BPSS1534	1.3	-1.0
BsaY	BPSS1535	2.3*	1.3
BsaX	BPSS1536	1.2	-1.2
BsaW	BPSS1537	1.2*	1.2
BsaV	BPSS1538	1.1	1.1
BsaU	BPSS1539	2.9*	1.0
BsaT	BPSS1540	1.6*	1.9*
BsaS	BPSS1541	1.6*	1.2
BsaR	BPSS1542	1.1	1.1
BsaQ	BPSS1543	1.2	1.1
BsaP	BPSS1544	2.4*	1.1
BsaO	BPSS1545	1.3	1.1
BsaN	BPSS1546	1.3	1.1
BsaL	BPSS1548	-1.1	1.3
BsaK	BPSS1549	1.1	1.2
**Translocator proteins**			
BipD	BPSS1529	1.8*	1.8*
BipC	BPSS1531	1.4*	1.4*
BipB	BPSS1532	1.3	1.3
**Effector proteins**			
BopB	BPSS1517	-1.2	1.0
BopA	BPSS1524	2.2*	1.8
BopE	BPSS1525	1.2	1.4*

* Genes showed mean significant differences comparing between standard LB medium (170 mM) and LB with 320 mM NaCl using *t*-test (*P value *< 0.05).

By looking at the transcription of *bsa*-encoded genes, we were able to establish that NaCl induces their expression. However it is possible that other T3SS effectors encoded elsewhere on the chromosome might be co-expressed with *bsa *genes in response to salt stress. To find other candidate T3SS effectors of *B. pseudomallei*, we used Self Organization Maps (SOM) based on the transcription profiles of the genes encoding the effectors BopA and BopE to identify 94 genes that had similar expression patterns (Additional file 4.) Among the co-regulated genes were other *bsa*-associated genes (e.g. those encoding BipB and the predicted chaperone BicP). Moreover, we also examined the direction and magnitude of transcription of predicted T3SS effectors that were previously proposed by Haraga *et al *[26] on the basis of homology with known effectors of other bacteria (Table 3 and Additional file 5). The results showed that only the T3SS-associated genes encoded within the *bsa *locus appeared to be significantly induced under salt stress (*bopA*, *bopE, bipC*, *bipB*, *bsaP*), with non-Bsa putative effectors apparently being insensitive to exogenous NaCl under the conditions tested. Thus, we did not find any other candidate T3SS effectors among the genes co-regulated with BopA and BopE, including those identified recently by Haraga *et al*. [27].

**Table 3:** Effect of NaCl on transcription of genes associated with homologs of known T3SS effectors in *B. pseudomallei *K96243 [27].

Putative function	Gene	Fold change	
		
		3 hrs	6 hrs
FG-GAP/YD repeat domain protein	BPSL0590	-1.2	-1.1
DNA polymerase III subunits gamma and tau	BPSL1498	1.2	1.0
Putative outer membrane protein	BPSL1631	-1.1	1.3
Hypothetical protein	BPSL1705	-1.0	1.0
Putative lipoprotein	BPSL1902	-1.2	-1.0
RND efflux system, outer membrane lipoprotein, NodT family protein	BPSL1972	1.2	-1.1
Putative exported phospholipase	BPSL2198	-1.0	1.1
Putative methyl-accepting chemotaxis protein	BPSL2367	-1.6	1.0
Putative prolin-rich exported protein	BPSL2472	-1.2	-1.1
Hypothetical protein	BPSL2699	-1.1	1.2

Hypothetical protein	BPSS0088	1.3	-1.1
Pentapeptide repeat family protein	BPSS0182	1.0	1.0
Hypothetical protein	BPSS0183	-1.1	1.2
Surface-exposed protein	BPSS0796	1.0	1.1
ATP/GTP binding protein	BPSS1385	-1.2	1.0
Tash protein PEST motif family	BPSS1434	-1.1	-1.0
Membrane-anchored cell surface protein	BPSS1439	-1.1	-1.0
Hypothetical protein	BPSS1504	1.2	1.3
Hypothetical protein	BPSS1505	1.1	1.1
BopA	BPSS1524	2.2	1.8
BopE	BPSS1525	1.2	1.4
BipC	BPSS1531	1.4	1.4
BipB	BPSS1532	1.3	1.3
BsaP	BPSS1544	2.4	1.1
Putative lipoprotein	BPSS1974	-1.0	1.1
Hypothetical protein	BPSS2063	-1.1	1.1
Hypothetical protein	BPSS2166	1.0	-1.2

### Validation of the differential transcription of *B. pseudomallei *genes by exogenous salt

**Figure 2:**
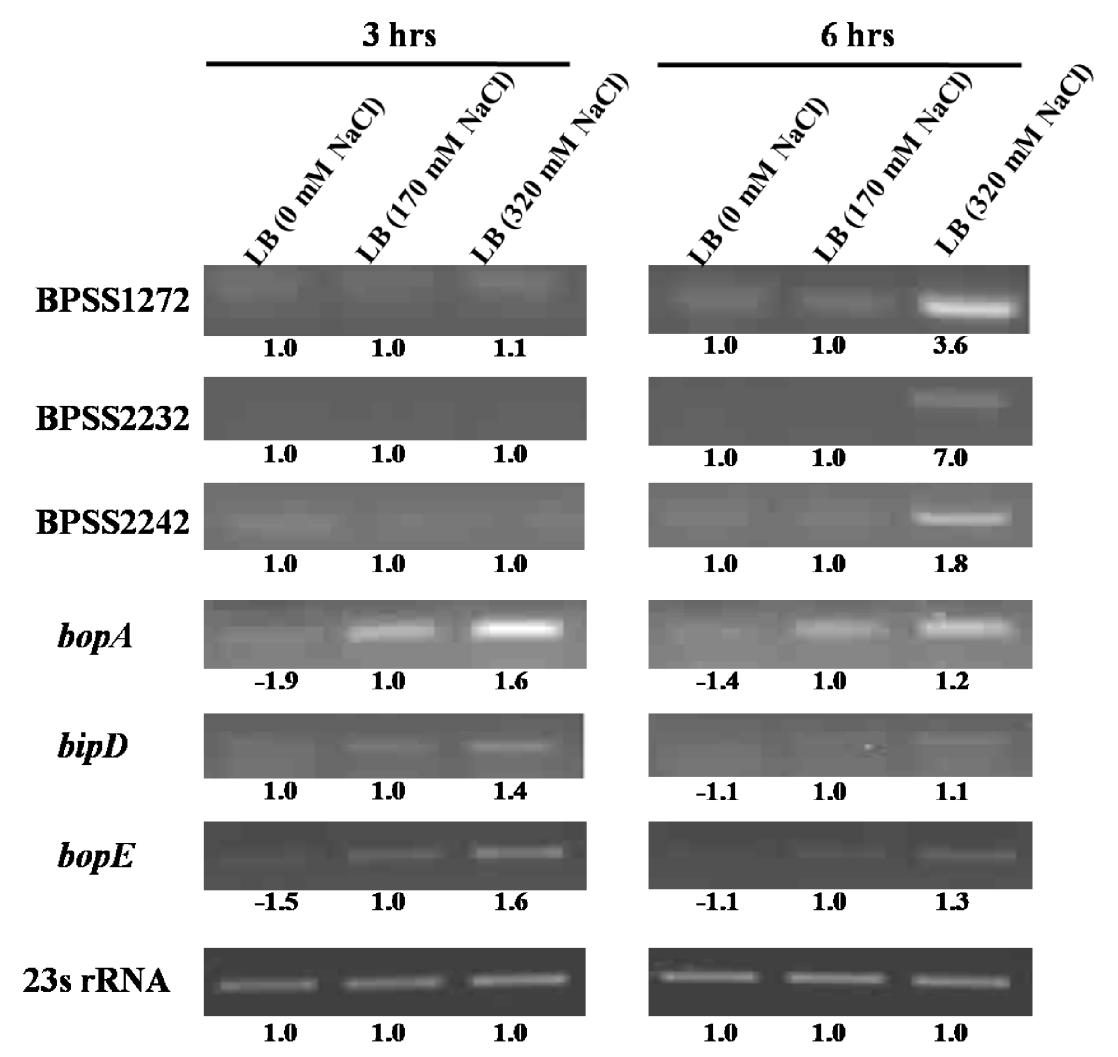
**Confirmation of microarray data by semiquantitative RT-PCR**. Each row represents an individual *B. pseudomallei *gene, and columns represent transcript levels in different media. The numbers below each gel image indicate the fold change of individual band intensities between a particular condition compared to standard LB medium containing 170 mM NaCl. 23 S rRNA expression is also shown (bottom row). The level of this control RNA was unchanged under the conditions examined. No products were amplified in the absence of reverse transcriptase, indicating that RNA samples were free of DNA.

To validate the differential transcription of genes observed by microarray analysis, selected transcripts were amplified by RT-PCR and band intensities quantified by densitometric analysis. The experiments were performed in duplicate using total RNA extracted from bacteria grown in salt-free LB, standard LB (170 mM NaCl) and LB containing 320 mM NaCl at 3 and 6 hrs post-inoculation. In all cases, RT-PCR analysis mirrored the timing and direction of change of transcription of the differentially transcribed genes identified by microarray analysis (Figure 2). In most cases the magnitude of the change was also comparable. Thus, up-regulation of BPSS2232, BPSS1272 and BPSS2242 (which respectively encode an Acyl-CoA dehydrogenase, a hypothetical protein and an oxidoreductase) was confirmed to occur at 6 hrs but not 3 hrs in the presence of added NaCl as found by microarray analysis (Table 1). Furthermore, the *bsa*-derived genes BPSS1529, BPSS1524, and BPSS1525 (which respectively encode the translocon component BipD and effectors BopA and BopE) were confirmed by RT-PCR to be upregulated in the presence of 320 mM NaCl (Figure 2). Increases for the *bsa*-derived genes occurred in a dose dependent manner, increasing from zero to 170 mM to 320 mM NaCl (Figure 2).

**Figure 3:**
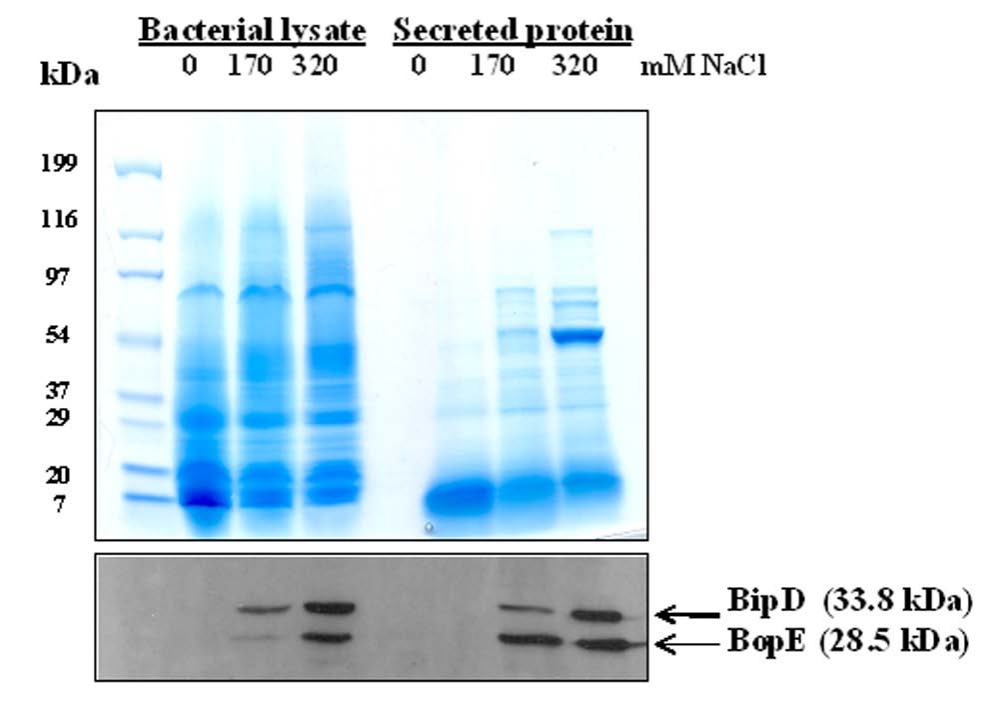
**BipD and BopE expression of *B. pseudomallei *cultured in LB medium with and without exogenous salt**. *B. pseudomallei *K96243 was cultured in LB broth supplemented with 0, 170, or 320 mM NaCl for 6 hrs. Bacterial lysate and secreted proteins were separated by 12% polyacrylamide gel and the blotted proteins were reacted with an anti-BipD and anti-BopE antibodies as described in the Methods. Molecular mass markers are shown on the left. Lanes 1-3 are bacterial cell lysates and lanes 4-6 are secreted proteins from culture supernatants.

The finding that genes encoding the Bsa T3SS were induced under high salinity was also reflected in protein levels. When *B. pseudomallei *K96243 was cultured in LB broth containing 320 mM NaCl, expression and secretion of the invasion-associated Type III secreted proteins BipD and BopE was enhanced when compared to standard LB, and in turn levels were higher than in salt-free medium (Figure 3). We observed a correlation between the increased expression of BopE and BipD from almost salt-free medium to higher levels of salt suggesting the importance of salt in the induction of the T3SS. These patterns of induction were also noted in an independent *B. pseudomallei *strain designated 10276 (data not shown) [28]. Taken together, these findings imply that expression of the Bsa T3SS of *B. pseudomallei *is enhanced by salt stress.

### Salt-stress increases invasion of host cells by *B. pseudomallei*

**Figure 4:**
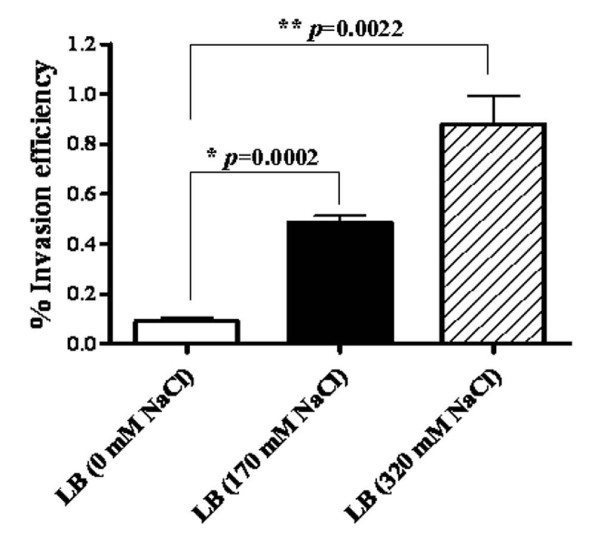
**Invasion of A549 epithelial cells by *B. pseudomallei***. A549 cells were infected with an overnight cultures of *B. pseudomallei *K96243 grown in in NaCl-free LB broth (open bar), LB broth with 170 mM NaCl (solid bar), or LB broth with 320 mM (striped bar). Intracellular bacteria were counted after lysing infected cells at 4 hrs-post-infections. Asterisks indicate significant differences (*P value *< 0.05, *t*-test) between groups. Error bars represent standard errors of the means for experiments performed in triplicate.

The ability of *B. pseudomallei *to invade non-phagocytic host cells is partly dependent on the Bsa T3SS [1,2] and is believed to contribute to the pathogenesis of melioidosis. Owing to the induction of *bsa *genes by exogenous salt, we investigated whether salt stress affects invasion of *B. pseudomallei *into A549 human lung respiratory epithelial cells. Overnight culture of *B. pseudomallei *in LB broth supplemented with NaCl (170 and 320 mM) led to significantly increased invasion into A549 cells relative to bacteria cultured in NaCl-free LB broth (*P value *= 0.0002 and 0.0022, respectively) (Figure 4). We additionally showed a significant difference in invasion capacity between *B. pseudomallei *cultured in LB with 170 and 320 mM NaCl (*P value *= 0.0272). The invasion efficiency of *B. pseudomallei *grown in NaCl-free LB was 0.09% in contrast to, those of salt-treated bacteria (0.49 and 0.88% in LB with 170 and 320 mM NaCl, respectively). To our knowledge this is the first report revealing that salinity affects the ability of *B. pseudomallei *to invade host cells. Although invasion was enhanced after overnight culture in salt-containing media, culturing *B. pseudomallei *in NaCl supplemented medium up to 320 mM for either 3 or 6 hrs did not significantly affect the ability of the bacteria to invade A549 cells (data not shown).

## Discussion

Alterations in NaCl content and therefore osmolarity in various environmental and host conditions are known conditions that most bacteria must counteract for survival [16]. At low concentrations, NaCl is necessary for bacterial growth, however at high concentrations it is capable of causing considerable stress and even cell death. *B. pseudomallei *is an environmental saprophyte that can survive and multiply under difficult environmental conditions [1,2]. It is likely therefore that *B. pseudomallei *must have the mechanisms to sense changes in osmolarity in the environment and host, and to modulate its gene expression accordingly.

We found that at high salt concentration (320 mM final concentration of NaCl), there was no significant impairment in *B. pseudomallei *growth over a 6 hr period. This finding is consistent with observations in *B. cenocepacia *indicating that it can tolerate medium containing up to 450 mM NaCl for 10 hrs [18]. In our study, two and eight genes were shown to be significantly up-regulated in *B. pseudomallei *grown in high salt for 3 and 6 hrs respectively, when compared with standard LB medium containing 170 mM NaCl. Of the 10 genes that show a salt-induced increase in transcription, 7 are clustered on chromosome 2, which is enriched in genes mediating *B. pseudomallei *adaptation and virulence [29]. Importantly, none of these genes were among the list of growth phase-regulated genes identified by microarray analysis of *B. pseudomallei *by Rodrigues *et al *[30]. This implies that the altered transcription levels detected in this study are a reflection of the salt stress and not impairment of growth.

Although highly stringent statistical analysis identified only a small number of transcriptionally salt-altered *B. pseudomallei *genes, our data did correlate with previous findings in other bacteria. Remarkably, it has been reported that an adenylate cyclase (CyaB) acts as an osmosensor in the Gram negative saprophytic bacterium *Myxococcus xanthus *[31]. We found a 1.5 fold increase in the expression of a *B. pseudomallei *K96243 adenylate cyclase gene (BPSL3054) during exposure to high salt for 3 hrs which decreased again later. We postulate therefore that adenylate cyclase might function as an osmosensor in *B. pseudomallei*, or be involved in the transmission of the signal. For the formyltetrahydrofolate deformylase-derived gene (BPSL0543) that was also upregulated at 3 hrs may function in the same manner. In addition, another study by Bhatt and Weingart [18] reported that an oxidoreductase encoding gene (*bsrA*) has been found to be regulated in response to increasing NaCl concentrations in *B. cenocepacia*. A putative oxidoreductase encoding gene (BPSS2242) in *B. pseudomallei *K96243 was also up-regulated (10 fold up at 6 hrs) under salt stress. However, the exact role that oxidoreductases play in adaptation to osmotic stress is still unknown.

A study into the salt stress response of *Azospirillum brasilense*, a Gram-negative nitrogen-fixing bacterium associated with various plants, found an increase in the expression levels of its Acyl-CoA dehydrogenase coding gene [32]. Several reports indicate that Acyl-CoA dehydrogenases are involved in the changes of bacterial membrane fluidity during salt tolerance [33,34]. Our study identified an increased level of expression of BPSS1272 also coding for Acyl-CoA dehydrogenase domain protein (around 4.4 fold at 6 hrs) suggesting that Acyl-CoA dehydrogenase may play a role in response to high salt stress. We hypothesise that this role may be in modulation of the membrane layer when *B. pseudomallei *encounters high salt.

As osmotic shock was found to increase expression of T3SS in various pathogens [19-21], we also sought to obtain information on the effect of salt on transcription of the T3SSs of *B. pseudomallei*. Much research has been carried out on the Bsa T3SS of *B. pseudomallei*, demonstrating its critical role in pathogenesis and more precisely in escaping the phagosome [24,28,35], but few substrates secreted by this system have been identified [28,35]. We used a two tailed unpaired *t*-test to identify genes significantly up-regulated at 3 hrs. Our finding that the *bsa*-derived genes, in particular those encoding secreted translocon and effector proteins, are upregulated in the presence of salt by both microarray and RT-PCR analysis mirrors the ability of exogenous NaCl to activate T3SS in other bacteria. T3SS genes encoding for structural components, translocators and effectors in *P. aeruginosa *were upregulated under steady-state hyperosmotic stress [19], as were *Salmonella *Typhimurium SPI-1 genes encoding T3SS-1 translocon proteins in the presence of exogenous NaCl [26]. Interestingly, by *t-test *we also found that *B. pseudomallei *grown in high salt upregulated genes encoding a beta-lactamase family protein (BPSS2119) and GroEL (BPSS0477). The increased expression of these genes correlates with the report of increased beta-lactamase family and GroEL proteins detection in the *B. pseudomallei *secretome under high salinity [17]. Conversely, none of *B. pseudomallei *genes encoded for within T3SS-1, T3SS-2, and other virulence factors (i.e., phospholipases, hemolysin and *Burkholderia *intracellular motility A) were altered under salt stress in our study (Additional file 3).

Previously, Moore *et al*. [36] demonstrated a functional link between the ability to assimilate L-arabinose and repression of the *bsa*-derived Type III secretion genes, which the authors found may account for the differential virulence of *ara*-plus and -minus biotypes. Moore *et al*. [36] also analysed the global transcriptome of *B. pseudomallei *in the presence or absence of the *ara *operon to identify genes that may be co-regulated with the *bsa *apparatus. It is noteworthy that *bsaN*, a predicted positive transcriptional regulator of the *bsa *genes is up-regulated 1.3 fold at 3 hrs in NaCl-supplemented medium (though not significant by *t*-test), and further studies will be required to unravel the role of *bsaN *and other regulators in salt induction of T3SS genes.

A recent study generated a list of putative T3SS effectors in *B. pseudomallei *by comparing predicted coding sequences to known bacterial effectors including *Salmonella *and *Shigella *effector proteins [27]. Our investigation could not detect the co-regulation of these putative effector genes, such as a putative proline-rich exposed protein and ATP/GTP binding protein, with respect to salt stress in contrast to secreted effectors encoded within the *bsa *locus.

In an attempt to identify genes that may be co-regulated with the virulence-associated Bsa system under salt stress, we used Self Organization Maps based on BopA and BopE expression to find 94 genes with similar expression patterns. These transcriptional changes showed an up-regulation of genes associated with various bacterial functions not only T3SS but also metabolism, stress response, and membrane transportation. One of these genes was the *bsa *T3SS translocator *bipB*, which is involved in *B. pseudomallei *survival within macrophages [35]. Likewise, we also found the up-regulation of the RpoE regulatory gene, *mucB*. The sigma factor E (RpoE) has previously been reported to play a role in the response to environmental stress tolerance such as hyperosmolarity in *B. pseudomallei *[37]. Recently, it has been suggested that RpoE and AlgR in *P. aeruginosa *may coordinate regulation of the T3SS and the alginate biosynthesis pathway [38]. Such a link between RpoE-regulating MucB and salt-induction of the Bsa system may exist in *B. pseudomallei*, but further studies will be required to investigate this.

The salt-induced transcription of the invasion- and virulence-associated genes *bipD *and *bopE*, which respectively encode a translocon component [24] and a guanine nucleotide exchange factor that subverts actin dynamics [28], was confirmed to result in increased production and secretion of the proteins by Western blotting using specific antisera. BipD and BopE protein expression increased in a gradient from 0 mM to 170 mM to 320 mM NaCl at both RNA and protein levels at both 3 and 6 hrs. This provides compelling evidence that the two genes are regulated by NaCl concentration. BipD and BopE both contribute to invasion of non-phagocytic cells [24,28] and mutation of *bipD *markedly impairs the virulence of *B. pseudomallei *following intranasal or intraperitoneal inoculation of inbred mice [22]. Consistent with induction of these genes, invasion of A549 cells was enhanced following overnight culture in salt-supplemented LB medium in a manner partly dependent on a functional Bsa system. Likewise, it has been reported in *Pseudomonas aeruginosa *under steady-state growth that high salt could induce the T3SS [18]. Therefore, it is possible that an overnight culture of *B. pseudomallei *could induce the T3SS and other factors that might contribute in increase invasion efficiency. Our result is in good agreement with a previous report that *S. typhi *cultured in 300 mM NaCl containing LB broth exhibited an increased secretion of invasion proteins (SipC, SipB and SipA) (*Zhao L et al*., 2001). Also, this salt-treated *S. typhi *became highly invasive toward both epithelial cells and M cell of rat Peyer's pathches (*Zhao L et al*., 2001).

### Conclusions

This study revealed that *B. pseudomallei *responds to high salt/osmolarity by modulating the transcription of specific genes. Most of identified genes are within chromosome 2. Among these are several loci that are known to contribute to the pathogenesis of melioidosis, including the invasion-associated Bsa T3SS.

## Methods

### Bacterial strains and growth kinetics

*B. pseudomallei *strain K96243 was cultured in LB broth at 37°C for 18 hrs. To determine *B. pseudomallei *growth kinetics under salt stress, optical density of cultures at various time points was recorded. In brief, overnight-cultured *B. pseudomallei *adjusted to OD_600 _0.5 was subcultured 1:500 into standard LB broth without or with supplementation of NaCl (Merck) to obtain a final concentration of 320-620 mM NaCl. Every 2 hrs after subculture, serial dilution was performed for colony forming unit counts (CFU).

### RNA preparation and microarray analysis

An overnight culture of *B. pseudomallei *K96243 was subcultured 1:10 into 10 mL LB broth containing 170 or 320 mM NaCl. Four biological replicates were generated and analysed. RNA was isolated from 3 and 6 hrs cultures of *B. pseudomallei *grown at 37°C by adding two volumes of RNAprotect bacterial reagent (QIAGEN) to one volume of bacterial culture and incubating for 5 min at room temperature. Subsequently, total RNA was extracted from bacterial pellets using Trizol (Invitrogen) according to the manufacturer's instructions and treated with DNase before use.

RNA (Cy3) and *B. pseudomallei *K96243 genomic DNA (Cy5) labeling were carried out as described in the standard RNA vs DNA labeling protocol [39]. After removal of excess dyes, labelled cDNA was competitively hybridized to *B. mallei/pseudomallei *microarrays version 2 (kindly supplied by the J. Craig Venter Institute) using a hybridization buffer containing 50% formamide (Sigma), 5× SSC (Ambion), 0.1% SDS (Ambion), and 0.1 mM Dithiothreitol solution (DTT) (Sigma) for 20 hrs at 42°C. After hybridization, the slide was gently agitated in prewarmed 55°C low stringency wash solution (2× SSC, 0.1% SDS, and 0.1 mM DTT) and immersed in a new prewarmed 55°C low stringency wash solution. Slides were further washed twice in medium stringency wash solution (0.1× SSC, 0.1% SDS, and 0.1 mM DTT). Finally, the slides were washed twice in high stringency wash solution (0.1× SSC and 0.1 DTT) and immersed several times in MilliQ/DI water before being allowed to spin dry. The washed slides were scanned using a GMS 418 Array Scanner (Genetic MicroSystems) and fluorescence was quantified using ImaGene v7.5 software (BioDiscovery). Analysis was carried out as previously described [39]. Each time point was normalized to the expression in LB broth without NaCl prior testing with statistical analysis.

### RT-PCR

The RNA extracts used in the microarray experiments were used to confirm the results obtained from microarray studies using the SuperScript III one-step RT-PCR system (Invitrogen). All genes were amplified using gene specific primer pairs (Table 4) using the following conditions: 95°C (for 45 s), 58°C (for 45 s), and 72°C (for 30 s) for 25 cycles. Amplification of the 23 S rRNA gene using 23 s F and 23 s R primers (Table 4) was included as a control. The experiments were performed in duplicate and analyzed for band intensity by densitometry using GeneSnap/GeneTools software (Syngene).

**Table 4:** Oligonucleotide primers used for RT-PCR.

Primer Names	Oligo Sequences (5'-3')	Purpose
BPSS2232 F	CGGACTTCGACACCGACGCGCTGA	Forward primer for BPSS2232
BPSS2232 R	CGTGTGCCAGTCGCTGCCCGCGTA	Reverse primer for BPSS2232
BPSS1272 F	GGCACGAAGGAAGTCATCAA	Forward primer for BPSS1272
BPSS1272 R	CGACGCAGTATCTCCAGCTC	Reverse primer for BPSS1272
BPSS2242 F	GTGAGCCGCTACGAGGAC	Forward primer for BPSS2242
BPSS2242 R	ACGCCCCAGTAGTTCGTATC	Reverse primer for BPSS2242
BopA F	GTATTTCGGTCGTGGGAATG	Forward primer for *bopA*
BopA R	GCGATCGAAATGCTCCTTAC	Reverse primer for *bopA*
BipD F	GGACTACATCTCGGCCAAAG	Forward primer for *bipD*
BipD R	ATCAGCTTGTCCGGATTGAT	Reverse primer for *bipD*
BopE F	CGGCAAGTCTACGAAGCGA	Forward primer for *bopE*
BopE R	GCGGCGGTATGTGGCTTC G	Reverse primer for *bopE*
23S F	TTTCCCGCTTAGATGCTTT	Forward primer for 23S rRNA
23S R	AAAGGTACTCTGGGGATAA	Reverse primer for 23S rRNA

### Preparation of total and secreted protein and Western blotting

An overnight-culture of *B. pseudomallei *grown in salt-free LB broth, was centrifuged and the bacteria washed in salt-free medium to remove secreted proteins. The OD_600 _was adjusted to 0.5 then the washed bacteria subcultured 1:10 into LB broth containing 0, 170 or 320 mM NaCl and incubated at 37°C for 6 hrs. After centrifugation, bacterial pellets were lysed with Laemmli buffer to release intracellular proteins. Secreted proteins were isolated from identical volumes of 0.45 μM-filtered supernatants from the centrifuged cultures by using Strataclean beads (Stratagene). The supernatants were confirmed to derive from cultures containing identical numbers of viable bacteria, therefore protein levels are not anticipated to reflect cell lysis. Proteins were separated by SDS polyacrylamide gel electrophoresis and transferred to PVDF membrane. The blotted membranes were probed with rabbit polyclonal antiserum against BopE [28] or BipD [24] and detected by horseradish peroxidase-conjugated donkey anti-rabbit IgG (GE Healthcare) and developed using a chemiluminescent substrate (ECL; GE Healthcare).

### Invasion assay

An invasion assay in the human respiratory epithelial cell line A549 was performed as described [25] with some modifications. Briefly, an A549 cell line was infected with overnight culture of *B. pseudomallei *in LB broth containing 0, 170 or 320 mM NaCl at a multiplicity of infection (MOI) of 50 for 3 hrs to bring bacteria in contact with the cells and allow bacterial entry. The monolayers were overlaid with a medium containing 250 μg/ml of kanamycin (Gibco) to kill extracellular bacteria for 1 hr. The viable intracellular bacteria were released from the infected cells at 4 hrs post-infection by lysis with 0.5% Triton X-100 (Sigma-Aldrich) and plated on Trypticase soy agar. Colony forming units were measured after 36-48 hrs of incubation at 37°C. The percentage invasion efficiency is calculated as the number of intracellular bacteria at 4 hrs post-infection divided by the CFU added × 100. All assays were conducted in triplicate and data from two independent experiments is presented.

### Statistical analysis

In the microarray analysis, the effect of salt on the magnitude of transcription of genes relative to control was tested for statistical significance using ANOVA with a 5% confidence interval and Benjamini-Hochberg multiple testing correction in GeneSpring (Silicon Genetics). Alternatively, an unpaired *t*-test was calculated for selected-gene groups at the 5% confidence interval in GraphPad Prism 4 program (Statcon). Results were considered significant at a *P value *of ≤ 0.05.

### Microarray data accession number

The complete microarray data set generated in this study is deposited for public access in the ArrayExpress under accession number E-MEXP-2302.

## Authors' contributions

PP and SK designed the research. PP and ES prepared the DNA/RNA samples for microarray and RT-PCR experiments. PP, RAS and JC carried out the microarray experiment and analysis. JMS performed the Western blotting. PP and VM carried out the invasion assay. PP, JC and SK wrote the manuscript. MPS and BWW critically revised the manuscript for its important intellectual content. All authors read and approved the final version of the manuscript.

## Acknowledgements

This work was partially supported by the Defense Science and Technology Laboratory (UK) and the Siriraj Grant for Research and Development (Thailand). PP was supported by Siriraj Graduate Scholarship and by the Royal Golden Jubilee Ph.D. Program (PHD0175/2548). We acknowledge the J. Craig Venter Institute for provision of *B. pseudomallei/mallei *microarrays.

## Supplementary Material

Additional file 1 **Cluster diagram of sample replicates in this study**. Standard correlation scores between microarray pairs are shown in white. Click here for file

Additional file 2 **The effect of NaCl on transcription of *bsa *T3SS genes in *B. pseudomallei *K96243 (presented in color graph)**. Click here for file

Additional file 3 **Effect of NaCl on transcription of selected genes associated with the T3SS-1, T3SS-2, and other virulence/non-virulence factors in *B. pseudomallei *K96243**. Click here for file

Additional file 4 **Ninety four genes identified using Self organization maps (SOM) showed expression patterns similar to *bopA *and *bopE *levels**. Click here for file

Additional file 5 **Effect of NaCl on transcription of genes encoding homologs of known T3SS effectors in *B. pseudomallei *K96243 (presented in color graph)**. Click here for file
