# Supplementary material for: Global transcriptional profiling of Burkholderia pseudomallei under salt stress reveals differential effects on the Bsa type III secretion system
Source: BMC Microbiol. 2010 Jun 14;10:171. doi: 10.1186/1471-2180-10-171 (PMC2896371; doi:10.1186/1471-2180-10-171)
Supplement: Additional file 1 — Cluster diagram of sample replicates in this study. Standard correlation scores between microarray pairs are shown in white. [file 1471-2180-10-171-S1.DOC]

**Additional file 1. Cluster diagram of sample replicates in this study. Standard correlation scores between microarray pairs are shown in white.**


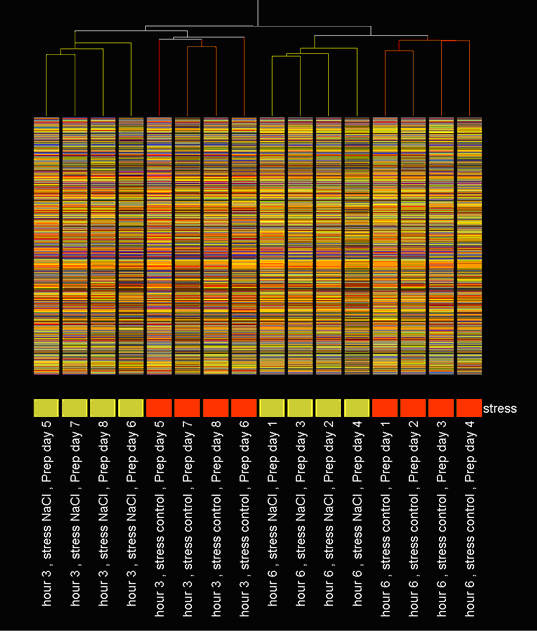


**0.88**

**0.92**

**0.87**

**0.87**

**0.86**

**0.91**

**0.88**

**0.91**

**0.91**

**0.87**

**0.91**

**0.86**
