# Supplementary material for: Global transcriptional profiling of Burkholderia pseudomallei under salt stress reveals differential effects on the Bsa type III secretion system
Source: BMC Microbiol. 2010 Jun 14;10:171. doi: 10.1186/1471-2180-10-171 (PMC2896371; doi:10.1186/1471-2180-10-171)

**Additional file 2. The effect of NaCl on transcription of *bsa* T3SS genes in *B. pseudomallei* K96243 (presented in color graph).**


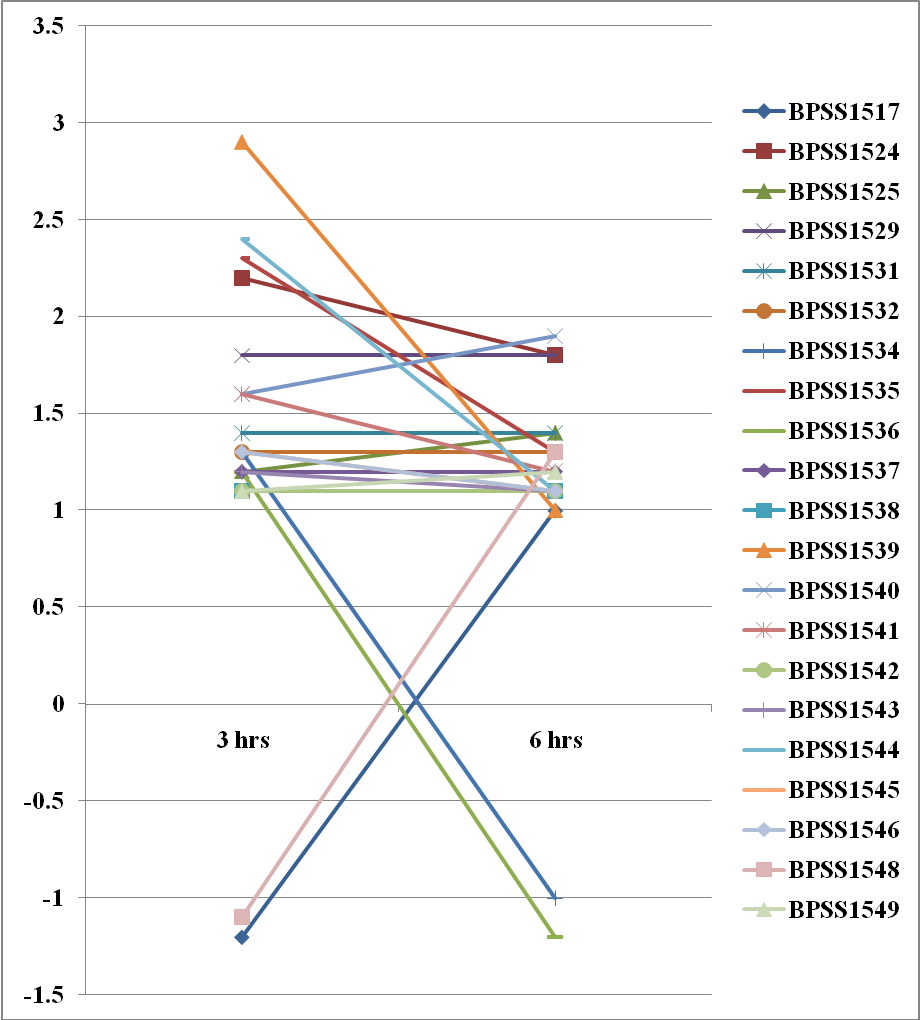

Supplement: Additional file 2 — The effect of NaCl on transcription of bsa T3SS genes in B. pseudomallei K96243 (presented in color graph). [file 1471-2180-10-171-S2.DOC]
