# Supplementary material for: Global transcriptional profiling of Burkholderia pseudomallei under salt stress reveals differential effects on the Bsa type III secretion system
Source: BMC Microbiol. 2010 Jun 14;10:171. doi: 10.1186/1471-2180-10-171 (PMC2896371; doi:10.1186/1471-2180-10-171)
Supplement: Additional file 3 — Effect of NaCl on transcription of selected genes associated with the T3SS-1, T3SS-2, and other virulence/non-virulence factors in B. pseudomallei K96243. [file 1471-2180-10-171-S3.DOC]

**Additional file 3. Effect of NaCl on transcription of selected genes associated with the T3SS-1, T3SS-2, and other virulence/non-virulence factors in *B. pseudomallei* K96243.**

| **Putative function** | **Gene** | **Fold change** | | ***P value*** |
| --- | --- | --- | --- | --- |
|  |  | **3 hrs** | **6 hrs** |  |
| **T3SS-1** |  |  |  |  |
|  | BPSS1390 | 1.1 | 1.0 | > 0.05 |
|  | BPSS1392 | 1.0 | 1.1 | > 0.05 |
|  | BPSS1393 | 1.1 | 1.1 | > 0.05 |
|  | BPSS1394 | 1.1 | 1.1 | > 0.05 |
|  | BPSS1395 | 1.1 | 1.1 | > 0.05 |
|  | BPSS1396 | 1.2 | 1.0 | > 0.05 |
|  | BPSS1397 | 1.1 | 1.1 | > 0.05 |
|  | BPSS1398 | 1.1 | 1.2 | > 0.05 |
|  | BPSS1399 | 1.1 | 1.1 | > 0.05 |
|  | BPSS1400 | 1.2 | 1.1 | > 0.05 |
|  | BPSS1401 | 1.1 | 1.1 | > 0.05 |
|  | BPSS1403 | 1.2 | 1.1 | > 0.05 |
|  | BPSS1404 | 1.1 | 1.1 | > 0.05 |
|  | BPSS1405 | 1.1 | 1.0 | > 0.05 |
|  | BPSS1407 | 1.1 | 1.2 | > 0.05 |
|  | BPSS1409 | 1.1 | 1.1 | > 0.05 |
| **T3SS-2** |  |  |  |  |
|  | BPSS1592 | 1.1 | 1.1 | > 0.05 |
|  | BPSS1603 | 1.3* | 1.1 | 0.0495 |
|  | BPSS1607 | 1.1 | 1.1 | > 0.05 |
|  | BPSS1612 | 1.1 | 1.1 | > 0.05 |
|  | BPSS1614 | 1.1 | 1.1 | > 0.05 |
|  | BPSS1616 | 1.2 | 1.0 | > 0.05 |
|  | BPSS1617 | -1.4* | 1.2 | 0.0098 |
|  | BPSS1618 | 1.2 | 1.0 | > 0.05 |

* Genes showed mean significant differences comparing between standard LB medium (170 mM) and LB with 320 mM NaCl using *t*-test (*P value* < 0.05).

**Additional file 3. Effect of NaCl on transcription of selected genes associated with the T3SS-1, T3SS-2, and other virulence/non-virulence factors in *B. pseudomallei* K96243.** (continued)

| **Putative function** | **Gene** | **Fold change** | | ***P value*** |
| --- | --- | --- | --- | --- |
|  |  | **3 hrs** | **6 hrs** |  |
| **T3SS-2 (continued)** |  |  |  |  |
|  | BPSS1620 | 1.1 | 1.0 | > 0.05 |
|  | BPSS1621 | 1.0 | 1.1 | > 0.05 |
|  | BPSS1622 | 1.1 | 1.1 | > 0.05 |
|  | BPSS1623 | 1.1 | 1.2 | > 0.05 |
|  | BPSS1624 | 1.1 | 1.1 | > 0.05 |
|  | BPSS1625 | 1.2 | 1.2 | > 0.05 |
|  | BPSS1626 | 1.1 | 1.1 | > 0.05 |
|  | BPSS1627 | 1.1 | 1.2 | > 0.05 |
|  | BPSS1628 | 1.1 | 1.1 | > 0.05 |
|  | BPSS1630 | 1.2 | 1.1 | > 0.05 |
| **T6SS** |  |  |  |  |
| BimA | BPSS1492 | 1.2 | 1.1 | > 0.05 |
| **Phospholipase** |  |  |  |  |
| PLC2 | BPSL0338 | 1.1 | 1.3 | > 0.05 |
| PLC1 | BPSL2403 | 1.1 | 1.2 | > 0.05 |
| **Hemolysin** |  |  |  |  |
|  | BPS30803 | 1.1 | 1.1 | > 0.05 |
| **Chaperone** |  |  |  |  |
| GroEL | BPSL2697 | 1.1 | 1.1 | > 0.05 |
| GroEL | BPSS0477 | 1.1 | 1.2* | 0.0159 |
| **Beta-lacmatase enzyme** |  |  |  |  |
|  | BPSL0374 | 1.1 | 1.1 | > 0.05 |
|  | BPSL1561 | 1.2 | 1.0 | > 0.05 |
|  | BPSL2708 | 1.1 | 1.2 | > 0.05 |

* Genes showed mean significant differences comparing between standard LB medium (170 mM) and LB with 320 mM NaCl using *t*-test (*P value* < 0.05).

**Additional file 3. Effect of NaCl on transcription of selected genes associated with the T3SS-1, T3SS-2, and other virulence/non-virulence factors in *B. pseudomallei* K96243.** (continued)

| **Putative function** | **Gene** | **Fold change** | | ***P value*** |
| --- | --- | --- | --- | --- |
|  |  | **3 hrs** | **6 hrs** |  |
| **Beta-lacmatase enzyme (continued)** |  |  |  |  |
|  | BPSL3272 | 1.2 | 1.0 | > 0.05 |
|  | BPSS0946 | 1.1 | 1.0 | > 0.05 |
|  | BPSS1915 | 1.0 | 1.1 | > 0.05 |
|  | BPSS1997 | 1.1 | 1.1 | > 0.05 |
|  | BPSS2119 | 1.1 | 1.2* | 0.0494 |
| **Capsule polysaccharide** |  |  |  |  |
|  | BPSL2787 | 1.2 | 1.2 | > 0.05 |
|  | BPSL2788 | 1.1 | 1.1 | > 0.05 |
|  | BPSL2789 | 1.1 | 1.2 | > 0.05 |
|  | BPSL2790 | 1.1 | 1.1 | > 0.05 |
|  | BPSL2791 | 1.2 | 1.1 | > 0.05 |
|  | BPSL2792 | 1.0 | 1.1 | > 0.05 |
|  | BPSL2793 | 1.2 | 1.1 | > 0.05 |
|  | BPSL2794 | 1.1 | 1.0 | > 0.05 |
|  | BPSL2795 | 1.1 | 1.3 | > 0.05 |
|  | BPSL2796 | 1.1 | 1.2 | > 0.05 |
|  | BPSL2797 | 1.0 | 1.0 | > 0.05 |
|  | BPSL2798 | 1.1 | 1.1 | > 0.05 |
|  | BPSL2799 | 1.1 | 1.0 | > 0.05 |
|  | BPSL2800 | 1.1 | 1.1 | > 0.05 |
|  | BPSL2801 | 1.1 | 1.0 | > 0.05 |
|  | BPSL2802 | 1.1 | 1.0 | > 0.05 |
|  | BPSL2803 | 1.1 | 1.1 | > 0.05 |

* Genes showed mean significant differences comparing between standard LB medium (170 mM) and LB with 320 mM NaCl using *t*-test (*P value* < 0.05).

**Additional file 3. Effect of NaCl on transcription of selected genes associated with the T3SS-1, T3SS-2, and other virulence/non-virulence factors in *B. pseudomallei* K96243.** (continued)

| **Putative function** | **Gene** | **Fold change** | | ***P value*** |
| --- | --- | --- | --- | --- |
|  |  | **3 hrs** | **6 hrs** |  |
| **Capsule polysaccharide (continued)** |  |  |  |  |
|  | BPSL2804 | 1.2 | 1.0 | > 0.05 |
|  | BPSL2805 | 1.1 | 1.2 | > 0.05 |
|  | BPSL2806 | 1.2 | 1.0 | > 0.05 |
|  | BPSL2807 | 1.1 | 1.1 | > 0.05 |
|  | BPSL2808 | 1.2 | 1.0 | > 0.05 |
|  | BPSL2809 | 1.1 | 1.2 | > 0.05 |
|  | BPSL2810 | 1.2 | 1.0 | > 0.05 |
