# Supplementary material for: Global transcriptional profiling of Burkholderia pseudomallei under salt stress reveals differential effects on the Bsa type III secretion system
Source: BMC Microbiol. 2010 Jun 14;10:171. doi: 10.1186/1471-2180-10-171 (PMC2896371; doi:10.1186/1471-2180-10-171)
Supplement: Additional file 4 — Ninety four genes identified using Self organization maps (SOM) showed expression patterns similar to bopA and bopE levels. [file 1471-2180-10-171-S4.DOC]

**Additional file 4. Ninety four genes identified using Self organization maps (SOM) showed expression patterns similar to *bopA* and *bopE*** levels.

| **Gene** | **Putative function** |
| --- | --- |
| BPSL0077 | hypothetical protein |
| BPSL0192 | alcohol dehydrogenase, zinc-containing |
| BPSL0200 | acetylglutamate kinase |
| BPSL0211 | lipid A biosynthesis lauroyl acyltransferase |
| BPSL0355 | putative inward rectifier potassium channel protein |
| BPSL0357 | sodium/hydrogen exchanger family protein |
| BPSL0427 | C4-dicarboxylate transport transcriptional regulatory protein |
| BPSL0616 | hypothetical protein |
| BPSL0656 | putative nucleotidyl transferase |
| BPSL0778 | hypothetical protein |
| BPSL0872 | MviN-like protein |
| BPSL0896 | hypothetical protein |
| BPSL1157 | putative phage integrase |
| BPSL1184 | putative sugar-related transport, membrane protein |
| BPSL1193 | conserved hypothetical protein |
| BPSL1203 | putative carbonic anhydrase |
| BPSL1210 | protein-export membrane protein |
| BPSL1222 | NADH dehydrogenase subunit L |
| BPSL1248 | hypothetical protein |
| BPSL1255 | hypothetical protein |
| BPSL1283 | Exodeoxyribonuclease V, beta subunit |
| BPSL1340 | hypothetical protein |
| BPSL1491 | 50S ribosomal protein L31 |
| BPSL1521 | hypothetical protein |
| BPSL1532 | putative ribosomal large subunit pseudouridine synthase D |
| BPSL1565 | putative MerR-family transcriptional regulator |
| BPSL1714 | putative threonine aldolase |
| BPSL1715 | argininosuccinate lyase |
| BPSL1782 | ferric iron reductase protein FhuF |
| BPSL1785 | hypothetical protein |
| BPSL1914 | putative multidrug resistance protein A |
| BPSL2098 | 2C-methyl-D-erythritol 2,4-cyclodiphosphate synthase |
| BPSL2187 | putative ATP-dependent RNA helicase |
| BPSL2218 | hypothetical protein |
| BPSL2225 | hypothetical protein |
| BPSL2434 | sigma factor algU regulatory protein MucB |

**Additional file 4. Ninety four genes identified using Self organization maps (SOM) showed expression patterns similar to *bopA* and *bopE* levels.** (continued)

| **Gene** | **Putative function** |
| --- | --- |
| BPSL2495 | radical SAM domain protein |
| BPSL2516 | cytidylate kinase |
| BPSLl2537 | disulfide bond formation protein B |
| BPSL2541 | guanine deaminase |
| BPSL2550 | hypothetical protein |
| BPSL2610 | ABC transporter, membrane permease |
| BPSL2617 | putative ABC-family amino acid transporter, ATP-binding component |
| BPSL2619 | hypothetical protein |
| BPSL2620 | MFS-family transport protein |
| BPSL2654 | putative ABC transport ATP-binding subunit |
| BPSL2824 | 3-methyl-2-oxobutanoate hydroxymethyltransferase |
| BPSL2836 | hypothetical protein |
| BPSL2904 | tyrosyl-tRNA synthetase |
| BPSL2935 | hypothetical protein |
| BPSL2940 | putative transport-related membrane protein |
| BPSL3055 | uracil-DNA glycosylase |
| BPSL3101 | 63 kDa protein |
| BPSL3193 | preprotein translocase SecY |
| BPSL3208 | 50S ribosomal protein L22 |
| BPSL3211 | 50S ribosomal protein L23 |
| BPSL3254B | hypothetical protein |
| BPSL3394 | acyl-CoA synthase |
| BPSS0130 | peptide synthase protein |
| BPSS0373 | hypothetical protein |
| BPSS0557 | putative amino acid transporter |
| BPSS0153 | glutamate/aspartate periplasmic binding protein precursor |
| BPSS0646 | hypothetical protein |
| BPSS0692 | fumarylacetoacetate (FAA) hydrolase family protein |
| BPSS0723 | hypothetical protein |
| BPSS0897 | short chain dehydrogenase |
| BPSS0928 | aliphatic sulfonates binding protein precursor |
| BPSS0943 | porin protein |
| BPSS1027 | RNA polymerase sigma-70 factor, ECF family |
| BPSS1169 | hypothetical protein |
| BPSS1170 | non-ribosomal peptide synthase/polyketide synthase |
| BPSS1234 | putative sugar ABC transporter, permease protein |

**Additional file 4. Ninety four genes identified using Self organization maps (SOM) showed expression patterns similar to *bopA* and *bopE* levels.** (continued)

| **Gene** | **Putative function** |
| --- | --- |
| BPSS1260 | hypothetical protein |
| BPSS1289 | hypothetical protein |
| BPSS1324 | D-cysteine desulfhydrase |
| BPSS1331 | phosphatase protein |
| BPSS1415 | 2-hydroxy-3-oxopropionate reductase |
| BPSS1417 | hypothetical protein |
| BPSS1419 | hydroxypyruvate isomerase |
| BPSS1446 | enoyl-CoA hydratase |
| BPSS1513 | hypothetical protein |
| BPSS1516 | hypothetical protein |
| BPSS1519 | Transposase |
| BPSS1523 | type III secretion chaperone BicP |
| BPSS1526 | invasion protein |
| BPSS1532 | BipB protein |
| BPSS1603 | secretion protein |
| BPSS1727 | hemagglutinin related protein |
| BPSS1173 | TubF protein |
| BPSS1913 | lysine-specific permease |
| BPSS2013 | hypothetical protein |
| BPSS2097 | hypothetical protein |
| BPSS2194 | hypothetical protein |
| BPSS2308 | hypothetical protein |
