# Supplementary material for: Global transcriptional profiling of Burkholderia pseudomallei under salt stress reveals differential effects on the Bsa type III secretion system
Source: BMC Microbiol. 2010 Jun 14;10:171. doi: 10.1186/1471-2180-10-171 (PMC2896371; doi:10.1186/1471-2180-10-171)
Supplement: Additional file 5 — Effect of NaCl on transcription of genes encoding homologs of known T3SS effectors in B. pseudomallei K96243 (presented in color graph). [file 1471-2180-10-171-S5.DOC]

**Additional file 5. Effect of NaCl on transcription of genes encoding homologs of known T3SS effectors in *B. pseudomallei* K96243 (presented in color graph).**

**
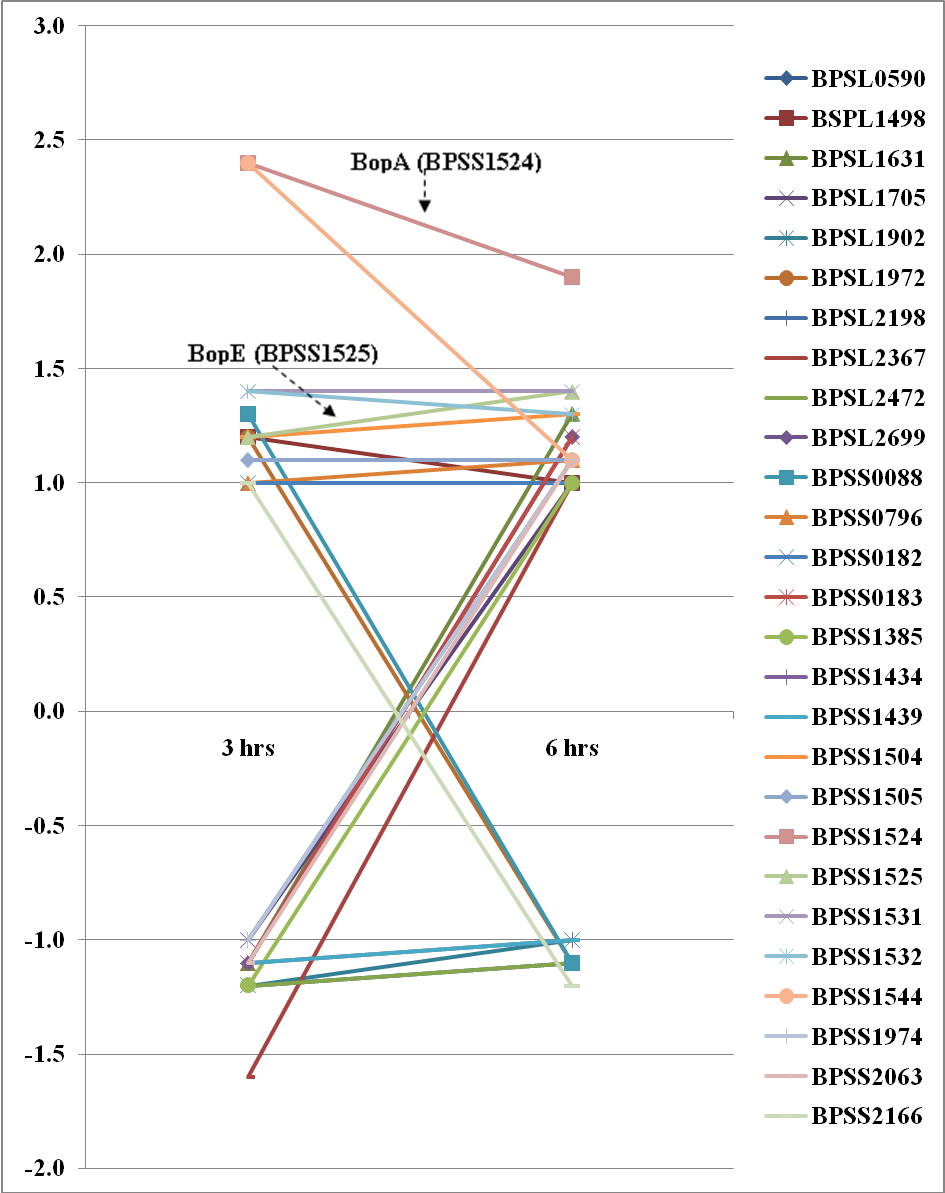
**
